# *Burkholderia thailandensis* Isolated from the Environment, United States

**DOI:** 10.3201/eid2903.221245

**Published:** 2023-03

**Authors:** Carina M. Hall, Nathan E. Stone, Madison Martz, Shelby M. Hutton, Ella Santana-Propper, Lora Versluis, Kieston Guidry, Marielisa Ortiz, Joseph D. Busch, Trevor Maness, Jonathan Stewart, Tom Sidwa, Jay E. Gee, Mindy G. Elrod, Julia K. Petras, Maureen C. Ty, Christopher Gulvik, Zachary P. Weiner, Johanna S. Salzer, Alex R. Hoffmaster, Sarai Rivera-Garcia, Paul Keim, Amanda Kieffer, Jason W. Sahl, Fred Soltero, David M. Wagner

**Affiliations:** Northern Arizona University, Flagstaff, Arizona, USA (C.M. Hall, N.E. Stone, M. Martz, S. Hutton, E. Santana-Propper, K. Guidry, J.D. Busch, P. Keim, J.W. Sahl, D.M. Wagner);; US Department of Agriculture, San Juan, Puerto Rico, USA (M. Ortiz, S. Rivera-Garcia, F. Soltero);; Texas Department of State Health Services, San Antonio, Texas, USA (T. Maness, J. Stewart, A. Kieffer);; Texas Department of State Health Services, Austin, Texas, USA (T. Sidwa);; Centers for Disease Control and Prevention, Atlanta, Georgia, USA (J.E. Gee, M.G. Elrod, J.K. Petras, M.C. Ty, C. Gulvik, Z.P. Weiner, J.S. Salzer, A.R. Hoffmaster)

**Keywords:** Burkholderia, Burkholderia thailandensis, Burkholderia pseudomallei, environmental pathogens, bacteria, water, waterborne infections, genomic islands, local adaptation, Western Hemisphere, Puerto Rico, Mississippi, Arkansas, Louisiana, Oklahoma, Texas, United States

## Abstract

*Burkholderia thailandensis*, an opportunistic pathogen found in the environment, is a bacterium closely related to *B. pseudomallei*, the cause of melioidosis. Human *B. thailandensis* infections are uncommon. We isolated *B. thailandensis* from water in Texas and Puerto Rico and soil in Mississippi in the United States, demonstrating a potential public health risk.

*Burkholderia thailandensis*, a gram-negative bacterium found in the environment, poses a public health threat both because of its ability to cause infections as an opportunistic pathogen and potential misidentification as the more pathogenic *B. pseudomallei*, its closest phylogenetic relative (*1*–*4*). *B. pseudomallei*, designated a Select Agent by the US Federal Select Agents Program and the causative pathogen of melioidosis, and *B. thailandensis* are found in the environment in some tropical regions, including Southeast Asia and northern Australia. *B. thailandensis*, a Biosafety Level 2 organism not classified as a Select Agent (*3*), has fewer safety restrictions than *B. pseudomallei*, and because it can be handled outside of Biosafety Level 3 laboratories, it is used by researchers as a surrogate in some experiments (*5*). In laboratory analyses, *B. thailandensis* is challenging to distinguish from *B. pseudomallei* because of their similar biochemical phenotypes, the only difference being that *B. thailandensis* can assimilate L-arabinose (*1*,*3*). *B. thailandensis* was described after researchers observed reduced virulence in an environmental isolate thought to be *B. pseudomallei*. Subsequent 16S rRNA gene analysis revealed a novel *Burkholderia* species named *B. thailandensis* after the geographic origin of the type strain (*3*).

Human *B. thailandensis* infections are uncommon (*1*,*4*), especially in the Western Hemisphere. Three previous clinical cases in that region have been reported, all from the southern United States: Louisiana in 1997, Texas in 2003 (*1*), and Arkansas in 2017 (*4*). Environmental sampling related to the 2003 case in Texas and previous environmental sampling for *B. pseudomallei* complex members did not recover *B. thailandensis* (*6*). *B. thailandensis* has been described primarily from the environment in Southeast Asia and Australia (*3*,*7*) and, recently, Africa (*8*). Occurrence of *B. thailandensis* in the environment in the Western Hemisphere remains poorly understood. We used a systematic approach to detect and isolate *B. thailandensis* from soil and water samples collected in Texas in November 2019 and November 2020 (*9*) and Puerto Rico during December 2018–March 2020.

## The Study

We collected 2,540 environmental samples, 370 (280 soil, 80 water, 10 environmental water tank scrapes) from Texas and 2,170 (1,650 soil, 520 water) from throughout Puerto Rico. From the collected samples, we detected *B. thailandensis* DNA in 10 complex broth samples, 4 from Texas and 6 from Puerto Rico (Appendix). Culturing (*10*) yielded *B. thailandensis* isolates from 5 samples, 1 from Texas and 4 from Puerto Rico. In addition, we isolated *B. thailandensis* from a soil sample collected in Mississippi in July 2022 during a melioidosis cluster investigation (*11*). Further, in 2021, we identified *B. thailandensis* infection in a 4th case-patient in the United States (Oklahoma) (Table). The patient was suspected to have aspirated water after a motor vehicle rollover into water; he died because of multiple complications (Appendix). 

**Table:** Genomes from global isolates used to generate whole-genome phylogeny in study of *Burkholderia thailandensis* from the environment in the United States*

Isolate	Alternative ID	Country (state/territory)	Sample type (source)	Year	MLST	GenBank accession no.
Bt10009†	165–01_P1_S7	USA (TX)	Environmental (water)	2019	1758	JALGJD00000000
Bt10013†	203–09_P1_S27	USA (PR)	Environmental (water)	2020	1772	JALGJC00000000
Bt9795†	61_10_S54_S1 copy3	USA (PR)	Environmental (water)	2018	1772	WCIR00000000
Bt9920†	89–06_P1_S1	USA (PR)	Environmental (water)	2018	1772	WCIQ00000000
Bt9942†	91–08_P2_S1	USA (PR)	Environmental (water)	2018	1772	WCIP00000000
BtMS2022a†		USA (MS)	Environmental (soil)	2022	2019	SRR22548212
BtOK2021a†		USA (OK)	Clinical (human)	2021	1772	SRR22548210
2.1		Vietnam	Environmental (soil)	2017	696	GCA_002803565.1
82172	34; 2002721621	France	Veterinary (horse)	1982	73	GCA_001555485.1
Bt4	49639	Australia	Environmental	Unknown	699	GCA_000170395.1
BtAR2017		USA (AR)	Clinical (human)	2017	101	GCA_004684955.1
E1		Papua New Guinea	Environmental	1995	669	GCA_001524325.1
E254		Thailand	Environmental (soil)	1992	345	GCA_000765375.1
E264	ATCC 700388	Thailand	Environmental (soil)	1994	80	GCA_003568605.1
E444	E0444	Thailand	Environmental (soil)	2002	79	GCA_000567945.1
E555		Cambodia	Environmental	2005	696	GCF_000179515.1
H0587	2002721121	USA (LA)	Clinical (human)	1997	101	GCA_000567905.1
MSMB59		Australia	Environmental (soil)	2006	669	GCA_001718595.1
MSMB60		Australia	Environmental (soil)	2006	669	GCA_001524345.1
Phuket 4W-1		Thailand	Environmental (water)	1965	80	GCA_000877335.1
TXDOH	CDC3015869; 2003015869	USA (TX)	Clinical (human)	2003	101	GCA_002888425.1
USAMRU Malaysia no. 20	2002721744	Malaysia	Unknown	Unknown	80	GCA_000706745.1

*Phylogeny shown in Figure. MLST, multilocus sequence type; USAMRU, US Army Medical Research Unit; TXDOH, Texas Department of Health. †Isolated in this study.

We used whole-genome analysis of those 7 isolates (National Center for Biotechnology Information BioProject nos. PRJNA575701, PRJNA818328, PRJNA908850) to place them within a larger phylogeographic context, including other *B. thailandensis* isolates from the United States and other global locations (Table; Figure). Environmental *B. thailandensis* isolates from Texas and Mississippi grouped in the same clade with clinical isolates from Texas and Louisiana and 2 environmental isolates from Asia. The 2021 clinical isolate from Oklahoma was most closely related to the isolate from the 2003 clinical case in Texas. Environmental isolates from Texas and Mississippi differed by more (4,639 single-nucleotide polymorphisms [SNPs]) than environmental isolates from Thailand and Australia (2,671 SNPs); *B. pseudomallei* isolates found in Australia and Asia are more diverse than isolates in the Americas (*10*). We observed little diversity among the 4 *B. thailandensis* isolates from Puerto Rico; total diversity was 62 SNPs, and distance between any 2 isolates was 28–36 SNPs.

**Figure:**
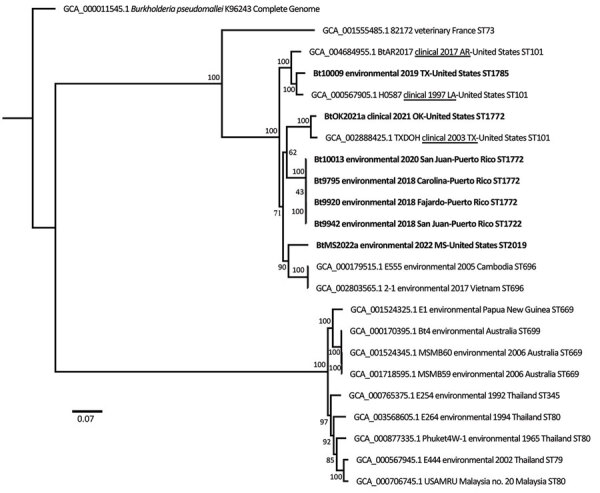
Whole-genome maximum-likelihood phylogeny of global isolates in study of *Burkholderia thailandensis* from the environment in the United States (Table). Tree was constructed with 1,000 bootstrap replicates and rooted with *B. pseudomallei*. Bold indicates *B. thailandensis* genomes generated from isolates collected in this study; other *B. thailandensis* from the Western Hemisphere have epidemiologic information underlined. Scale bar indicates 3,000 SNPs.

Among the isolates identified in our study, in silico multilocus sequence type analysis (https://pubmlst.org/organisms/burkholderia-pseudomallei) revealed novel *ace* allele 106 in the 4 isolates from Puerto Rico and the clinical isolate from Oklahoma, assigning all 5 to novel sequence type (ST) 1772. Novel *gltB* allele 175 was identified in the isolate from Texas, which was assigned to novel ST1785. The isolate from Mississippi, which had a unique combination of alleles, was assigned to novel ST2019.

## Conclusions

Our study confirms *B. thailandensis* endemicity in the environment in the United States, albeit of rare occurrence and low abundance, requiring extensive sampling to detect; we found *B. thailandensis* at only 3.7% of collection sites in Puerto Rico and 8% in Texas. However, the pathogen could be present in other unsampled areas in the southern United States and Puerto Rico. Substantial culturing was required to isolate bacteria from PCR-positive samples, suggesting low abundance or its presence being outcompeted by other bacteria. *B. thailandensis* abundance might vary seasonally or on the basis of precipitation levels. 

We detected *B. thailandensis* in Texas and Puerto Rico only from water samples, although they comprised only 24% of total (water and soil) samples collected at positive sites; all soil samples were negative for *B. thailandensis*. In contrast, in Thailand, *B. thailandensis* is most commonly isolated from soil (*12*). All 4 clinical cases from the United States were associated with traumatic injuries (*1*,*4*), 3 involving water (*1*), demonstrating the public health risk for disease from traumatic injuries related to contaminated water. This risk is especially relevant in Puerto Rico where *B. thailandensis* was detected within neighborhoods of the largest city, San Juan. Puerto Rico and the southeastern United States are prone to hurricane-induced flooding, which could increase the risk for infection by both *B. thailandensis* and *B. pseudomallei* (*13*).

Although samples were collected from 3 municipalities in northeastern Puerto Rico during a 1-year period, we found little phylogenetic diversity among the isolates, suggesting *B. thailandensis* may be widespread but rare in the environment in Puerto Rico and the result of a single introduction, as previously suggested for *B. pseudomallei* in Puerto Rico (*10*). We found evidence of possible local adaptation in Puerto Rico, which supports this hypothesis. We identified 113 genes unique to *B. thailandensis* isolates from Puerto Rico (Appendix), many of them potentially colocated in genomic islands, a pattern similar to one previously observed among *B. pseudomallei* isolates from Puerto Rico (*10*). Of note, 2 genes common to all *B. thailandensis* from Puerto Rico were present in some *B. pseudomallei* isolates from Puerto Rico but absent from all other global *B. pseudomallei* genomes (Appendix). In contrast, thousands of SNPs were found among *B. thailandensis* strains in the continental United States (Arkansas, Louisiana, Mississippi, Oklahoma, and 2003 clinical and 2019 environmental isolates from Texas). This finding suggests a long-term but cryptic presence of *B. thailandensis* in the southern United States, perhaps in water. It is unknown how long *B. thailandensis* can persist in water, but *B. pseudomallei* can survive in water for >16 years without nutrients (*14*).

Our study provides valuable information regarding *B. thailandensis* occurrence and the potential of water to serve as a reservoir and source of infection for this opportunistic pathogen in the southern United States and Puerto Rico, especially following flooding events. Because likely autochthonous melioidosis cases also have been reported from Texas (*15*), Puerto Rico (*10*), and Mississippi (*11*), clinicians should be aware of the potential of misidentifying *B. thailandensis* as *B. pseudomallei* because of their morphologic and biochemical similarities. 

Appendix Additional information about *Burkholderia thailandensis* found in the environment in the United States.
