# Supplementary material for: Burkholderia thailandensis Isolated from the Environment, United States
Source: Emerg Infect Dis. 2023 Mar;29(3):618–21. doi: 10.3201/eid2903.221245 (PMC9973702; doi:10.3201/eid2903.221245)
Supplement: Appendix — Additional information about Burkholderia thailandensis found in the environment in the United States. [file 22-1245-Techapp-s1.pdf]

Article DOI: https://doi.org/10.3201/eid2903.221245

EID cannot ensure accessibility for supplementary materials supplied by authors. Readers who have difficulty accessing supplementary content should contact the authors for assistance.

Burkholderia thailandensis Isolated from the Environment, United States

Appendix

Additional Methods

Environmental sampling was conducted in Puerto Rico and Texas using methods previously described (*1*,*2*) and were adapted from international consensus guidelines (*3*) with additional modifications developed by the Menzies School of Health and Research in Darwin, Australia (*4*). Permission was obtained from landowners to collect soil and/or water samples on their property and, when necessary, permits were received to collect from reserve lands. Briefly, at each site, we collected 10 to 100 soil samples at a depth of 30 cm from holes spaced 2.5 meters apart in one to two 10-hole transects or in a grid for each site as previously described (*1*). Water samples (150 mL) were collected along a linear transect with 2.5 meters between each sampling location, when possible, using one of two sampling approaches: For approach A water samples were collected (20 samples per site in Puerto Rico and 10 samples per site in Texas) approximately 1 meter from the shoreline, avoiding flowing water, whereas for approach B water samples were collected (10 samples per site in Puerto Rico) at five sampling locations with the first sample collected directly on the water's edge and another sample collected 1 meter from the shoreline (*5*). All water samples were filtered the day of collection using a Sartorius water filtration manifold with 0.22 µm nitrocellulose filters, as previously described (*1*). Environmental scrapes were only collected in Texas at one site from a partially empty residential 500-gallon water holding tank. Briefly, the bottom and sides of the tank were scraped, and the scrape contents were placed into a sterile 2 mL screw-cap tube (*2*).

A total of 2,170 environmental samples were collected throughout Puerto Rico during the months of December 2018, February and March 2019, and February and March 2020. These environmental samples consisted of 1,650 soil samples collected from 92 sites and 520 water

samples collected from 42 sites (300 water samples were collected from 20 sites using sampling approach A and 220 collected from 22 sites using sampling approach B). As previously described (*2*), a total of 210 environmental samples were collected in Atascosa County in Texas during November 2019, including 120 soil samples collected from eight sites, 80 water samples from eight sites, and 10 environmental scrapes from one site. Another 160 soil samples were collected from eight sites in Guadalupe, Goliad, and Wilson counties in Texas during November 2020 (*2*). In summary, samples were collected from 159 sites (25 from Texas and 134 from Puerto Rico) with *B. thailandensis* detected from seven sites (two from Texas and five from Puerto Rico) and isolated from five sites (one from Atascosa County in Texas [Bt10009] and four from the municipalities of Carolina [Bt9795], Fajardo [Bt9920], and San Juan [Bt9942, Bt10009] in Puerto Rico).

All collected samples were kept from direct UV exposure and shipped at ambient temperature to NAU for further processing. Upon arrival, samples were stored in the dark at ambient temperature except for the sampling approach B water filters, which were stored at 4°C. To prepare the environmental scrapes for culturing, the 2 mL tubes containing the scrapes were first vortex at high speed for 1 minute and then sonicated for 5 minutes using a Branson sonicator bath set to 70W, 42kHz at room temperature.

All samples were processed for detection and isolation of *Burkholderia* spp. as previously described (*1*), with the following modifications. Each water sample was filtered onto one filter, which was cut in half and only one half was used for the inoculation of 30 mL of Ashdown's broth. The entire contents of the environmental scrape were transferred to the 30 mL of Ashdown's broth. The soil was processed in the same way as Hall *et al.*, 2022 (*2*). Also, a *B. thailandensis* specific TaqMan assay (21-thai_all_110625) was used for molecular detection of a *B. thailandensis* DNA signal within DNA extractions, which were performed on 1 mL of the complex Ashdown's broth. Methods for the DNA extractions can be found in Hall *et al.*, 2019 (*1*).

The primers and probe used for the *B. thailandensis* specific TaqMan assay (21-thai_all_110625) were developed based on whole genome analysis of 1,130 *Burkholderia* genomes (*6*). The *B. thailandensis* TaqMan specific primers Bt_A_0625_961_F (5'-GTGCGCATCAGTATGGTCGT-3') and Bt_A_0625_1034_R (5'-

TGAGAGGCAAAACGAACGAA-3') and probe Bt_A_0625_1006_RP (5'-FAM-GCATCGCGGCAAGGTTGCTT-MGB-3') were used with the following assay conditions: a 10 µL PCR reaction containing the final concentrations of 1x Applied Biosystems TaqMan® Environmental Master Mix 2.0, 0.2 µM of each primer, 0.1 µM of the probe, 0.66 M of betaine, and 1 µL of undiluted DNA template. PCRs were run on the Applied Biosystems QuantStudio™ 7 (or 12K) Flex Real-Time PCR Systems with the following PCR conditions: 2 minutes at 50°C, 10 minutes at 95°C, and 35 cycles of 15 seconds at 95°C and 1 minute at 63°C. All samples were screened in duplicate.

If a *B. thailandensis* DNA signal was detected in an Ashdown's broth sample, isolation efforts were focused on that sample; culturing methods are described in Hall *et al.*, 2019 (*1*). Once a pure *B. thailandensis* isolate was obtained after at least three isolation streaks, long term glycerol stocks were created and high molecular weight gDNA was obtained using a QIAGEN DNeasy Blood & Tissue kit, following the Gram-Positive protocol in the manufacturer's instructions. Whole genome sequencing was performed as previously described (*1*).

Environmental sampling (soil, water, and plant material) was conducted in Mississippi in July of 2022 using methods previously described (*1*) and adapted from international consensus guidelines (*3*). Samples were shipped to the CDC in Atlanta, GA for culturing. Culturing of the environmental samples occurred as previously described (*1*), except Galimand's (TBSS-50) was used for the enrichment broth instead of Ashdown's broth. Colony morphologies resembling *B. pseudomallei* and *B. thailandensis* were selected as described in the consensus guidelines (*3*). Since the colony morphology of *B. pseudomallei* and *B. thailandensis* are very similar, arabinose agar and a Laboratory Response Network (LRN) species-specific PCR was used to identify the *B. thailandensis* isolate from Mississippi.

The 2021 Oklahoma clinical isolate of *B. thailandensis* was isolated from a 46-year-old male who presented to an emergency department in Oklahoma after a motor vehicle crash with a rollover into a creek near the Tulsa area with suspected water aspiration from the creek. The patient had no travel history and due to multiple complications did not survive. The *B. thailandensis* isolate was sent to the CDC in Atlanta, GA.

Multi-Locus Sequence Type (MLST)

In silico MLST analysis with FastMLST v0.0.15 (*7*) revealed a novel *ace* allele (106) in all four *B. thailandensis* isolates from Puerto Rico and in the 2021 Oklahoma clinical isolate, assigning all five to novel ST1772. A novel *gltB* allele (175) was identified in the single isolate from Texas assigning it to a novel ST1785. The novel ST for Texas was assigned as ST1785 (*ace*=106, *gltB*=5*, gmhD*=9, *lepA*=11, *lipA*=14, *narK*=20*,* ndh=14), the novel ST for Puerto Rico and Oklahoma were assigned as ST1772 (*ace*=106, *gltB*=5, *gmhD*=9, *lepA*=11, *lipA*=14, *narK*=20, *ndh*=14), and the Mississippi isolate had a unique combination of described MLST alleles and assigned as ST2019 (*ace*=6, *gltB*=5, *gmhD*=9, *lepA*=5, *lipA*=14, *narK*=20, *ndh*=14). Both ST1785 and ST1772 have all the same alleles as ST101, which was assigned to the Arkansas, Louisiana, and Texas *B. thailandensis* clinical samples, except for the *gltB* and *ace* difference described above.

Phylogenetic analyses

Genomes sequenced in this study were assembled with SPAdes v3.13.0 (*8*) using default parameters. All genome assemblies were aligned against *B. thailandensis* E254 (GCA_000765375.1) with NUCmer v3.1 (*9*) and single nucleotide polymorphisms (SNPs) were called with NASP v1.2.0 (*10*). SNPs that fell within duplicated regions, based on a reference self-alignment with NUCmer, were filtered from all downstream analyses. SNPs were also removed if they were <5 positions apart in the reference genome. SNP distances between genomes was determined with snp-dists v0.8.2 (https://github.com/tseemann/snp-dists) and variable sites were extracted with snp-sites v2.5.1(PMID:28348851). SNPs that had a valid nucleotide call in all query genomes (*n*=44,187 SNPs) were extracted from the outgroup genome, *B. pseudomallei* K96243 (*11*), and concatenated into a single multifasta file. A maximum-likelihood phylogeny was inferred on the concatenated alignment with IQ-TREE v1.6.12 (*12*), using the TVM+F+ASC+G4 substitution model with 1,000 bootstrap replicates (*13*).

Comparative genomics

To identify gene differences in genomes sequenced in this study, all genomes were annotated with Prokka v1.14.6 (PMID:24642063) and the pan-genome was calculated with

Panaroo v1.3.0 (PMID:32698896). The pan-genome was then aligned against all *B. thailandensis* genomes (n=22) with LS-BSR (large-scale blast score ratio) v1.2.3 (*14*) and BLAT v36x2 (*15*). Coding region sequences (CDSs) were identified that had a BSR value ≥0.9 in Puerto Rican isolates an a BSR value <0.4 in all other *B. thailandensis* genomes.

The 113 genes unique to the *B. thailandensis* isolates from Puerto Rico are listed in S1 Table. The two genes unique to all *B. thailandensis* from Puerto Rico, which also are present in some *B. pseudomallei* isolates from Puerto Rico but absent from all other global *B. pseudomallei* genomes, have locus tags MTQ99_16735 and MTQ99_16745.

References

1. Hall CM, Jaramillo S, Jimenez R, Stone NE, Centner H, Busch JD, et al. *Burkholderia pseudomallei*, the causative agent of melioidosis, is rare but ecologically established and widely dispersed in the environment in Puerto Rico. PLoS Negl Trop Dis. 2019;13:e0007727. PubMed https://doi.org/10.1371/journal.pntd.0007727

2. Hall CM, Romero-Alvarez D, Martz M, Santana-Propper E, Versluis L, Jiménez L, et al. Low risk of acquiring melioidosis from the environment in the continental United States. PLoS One. 2022;17:e0270997. PubMed https://doi.org/10.1371/journal.pone.0270997

3. Limmathurotsakul D, Dance DA, Wuthiekanun V, Kaestli M, Mayo M, Warner J, et al. Systematic review and consensus guidelines for environmental sampling of *Burkholderia pseudomallei.* PLoS Negl Trop Dis. 2013;7:e2105. PubMed https://doi.org/10.1371/journal.pntd.0002105

4. Kaestli M, Mayo M, Harrington G, Watt F, Hill J, Gal D, et al. Sensitive and specific molecular detection of *Burkholderia pseudomallei*, the causative agent of melioidosis, in the soil of tropical northern Australia. Appl Environ Microbiol. 2007;73:6891–7. PubMed https://doi.org/10.1128/AEM.01038-07

5. Stone NE, Hall CM, Ortiz M, Hutton SM, Santana-Propper E, Celona KR, et al. Diverse lineages of pathogenic *Leptospira* species are widespread in the environment in Puerto Rico, USA. PLoS Negl Trop Dis. 2022;16:e0009959. PubMed https://doi.org/10.1371/journal.pntd.0009959

6. Sahl JW, Vazquez AJ, Hall CM, Busch JD, Tuanyok A, Mayo M, et al. The effects of signal erosion and core genome reduction on the identification of diagnostic markers. MBio. 2016;7:7. PubMed https://doi.org/10.1128/mBio.00846-16

7. Guerrero-Araya E, Muñoz M, Rodríguez C, Paredes-Sabja D. FastMLST: a multi-core tool for multilocus sequence typing of draft genome assemblies. Bioinform Biol Insights. 2021;15:11779322211059238. PubMed https://doi.org/10.1177/11779322211059238

8. Bankevich A, Nurk S, Antipov D, Gurevich AA, Dvorkin M, Kulikov AS, et al. SPAdes: a new genome assembly algorithm and its applications to single-cell sequencing. J Comput Biol. 2012;19:455–77. PubMed https://doi.org/10.1089/cmb.2012.0021

9. Delcher AL, Salzberg SL, Phillippy AM. Using MUMmer to identify similar regions in large sequence sets. Curr Protoc Bioinformatics. 2003;Chapter 10:Unit 10 3.

10. Sahl JW, Lemmer D, Travis J, Schupp JM, Gillece JD, Aziz M, et al. NASP: an accurate, rapid method for the identification of SNPs in WGS datasets that supports flexible input and output formats. Microb Genom. 2016;2:e000074. PubMed https://doi.org/10.1099/mgen.0.000074

11. Holden MT, Titball RW, Peacock SJ, Cerdeño-Tárraga AM, Atkins T, Crossman LC, et al. Genomic plasticity of the causative agent of melioidosis, *Burkholderia pseudomallei.* Proc Natl Acad Sci U S A. 2004;101:14240–5. PubMed https://doi.org/10.1073/pnas.0403302101

12. Nguyen LT, Schmidt HA, von Haeseler A, Minh BQ. IQ-TREE: a fast and effective stochastic algorithm for estimating maximum-likelihood phylogenies. Mol Biol Evol. 2015;32:268–74. PubMed https://doi.org/10.1093/molbev/msu300

13. Kalyaanamoorthy S, Minh BQ, Wong TKF, von Haeseler A, Jermiin LS. ModelFinder: fast model selection for accurate phylogenetic estimates. Nat Methods. 2017;14:587–9. PubMed https://doi.org/10.1038/nmeth.4285

14. Sahl JW, Caporaso JG, Rasko DA, Keim P. The large-scale blast score ratio (LS-BSR) pipeline: a method to rapidly compare genetic content between bacterial genomes. PeerJ. 2014;2:e332. PubMed https://doi.org/10.7717/peerj.332

15. Kent WJ. BLAT—the BLAST-like alignment tool. Genome Res. 2002;12:656–64. PubMed

Appendix Table. Locus tags for 113 genes unique to *B. thailandensis* isolates from Puerto Rico

Unique locus tag
MTQ99_08690
MTQ99_14570
MTQ99_14575
MTQ99_14580
MTQ99_14585
MTQ99_14590
MTQ99_14595
MTQ99_14600
MTQ99_14605
MTQ99_14610
MTQ99_14615
MTQ99_14625
MTQ99_14630
MTQ99_14635
MTQ99_14640
MTQ99_14645
MTQ99_14650
MTQ99_14655
MTQ99_14660
MTQ99_14665
MTQ99_14670
MTQ99_14675
MTQ99_14680
MTQ99_15800
MTQ99_15805
MTQ99_15810
MTQ99_15815
MTQ99_15820
MTQ99_15825
MTQ99_15830
MTQ99_16730
MTQ99_16735
MTQ99_16740
MTQ99_16745
MTQ99_16750
MTQ99_16755
MTQ99_16760
MTQ99_16770
MTQ99_16775
MTQ99_16825
MTQ99_16830
MTQ99_16835
MTQ99_16840
MTQ99_16845
MTQ99_16850
MTQ99_16855
MTQ99_16860
MTQ99_16865
MTQ99_16870
MTQ99_16875
MTQ99_16885
MTQ99_16890
MTQ99_16895
MTQ99_16900
MTQ99_16905
MTQ99_16910
MTQ99_16920
MTQ99_16925
MTQ99_16930
MTQ99_16935
MTQ99_17175
MTQ99_17180
MTQ99_17185
MTQ99_26310

Unique locus tag
MTQ99_26315
MTQ99_26320
MTQ99_26325
MTQ99_26330
MTQ99_26335
MTQ99_26340
MTQ99_26345
MTQ99_26350
MTQ99_26355
MTQ99_26360
MTQ99_26375
MTQ99_26380
MTQ99_26385
MTQ99_26395
MTQ99_26445
MTQ99_26455
MTQ99_26460
MTQ99_26465
MTQ99_26470
MTQ99_26475
MTQ99_26480
MTQ99_26485
MTQ99_26750
MTQ99_26755
MTQ99_26760
MTQ99_26765
MTQ99_26770
MTQ99_26775
MTQ99_26785
MTQ99_26790
MTQ99_26795
MTQ99_26800
MTQ99_26805
MTQ99_26810
MTQ99_26815
MTQ99_26820
MTQ99_26825
MTQ99_27230
MTQ99_27235
MTQ99_27240
MTQ99_27480
MTQ99_27490
MTQ99_27495
MTQ99_28550
MTQ99_28630
MTQ99_28635
MTQ99_28640
MTQ99_28645
MTQ99_28650
